# Modelling [^18^F]LW223 PET data using simplified imaging protocols for quantification of TSPO expression in the rat heart and brain

**DOI:** 10.1007/s00259-021-05482-1

**Published:** 2021-08-02

**Authors:** Mark G. MacAskill, Catriona Wimberley, Timaeus E. F. Morgan, Carlos J. Alcaide-Corral, David E. Newby, Christophe Lucatelli, Andrew Sutherland, Sally L. Pimlott, Adriana A. S. Tavares

**Affiliations:** 1 University/ BHF Centre for Cardiovascular Science, University of Edinburgh, Edinburgh, UK; 2 Edinburgh Imaging, University of Edinburgh, Edinburgh, UK; 3 Centre for Clinical Brain Sciences, University of Edinburgh, Edinburgh, UK; 4 School of Chemistry, University of Glasgow, Glasgow, UK; 5 West of Scotland PET Centre, NHS Greater Glasgow and Clyde, Glasgow, UK

**Keywords:** TSPO, PET, Myocardial infarction, Kinetic modelling

## Abstract

**Purpose:**

To provide a comprehensive assessment of the novel 18 kDa translocator protein (TSPO) radiotracer, [^18^F]LW223, kinetics in the heart and brain when using a simplified imaging approach.

**Methods:**

Naive adult rats and rats with surgically induced permanent coronary artery ligation received a bolus intravenous injection of [^18^F]LW223 followed by 120 min PET scanning with arterial blood sampling throughout. Kinetic modelling of PET data was applied to estimated rate constants, total volume of distribution (*V*_*T*_) and binding potential transfer corrected (*BP*_*TC*_) using arterial or image-derived input function (IDIF). Quantitative bias of simplified protocols using IDIF versus arterial input function (AIF) and stability of kinetic parameters for PET imaging data of different length (40–120 min) were estimated.

**Results:**

PET outcome measures estimated using IDIF significantly correlated with those derived with invasive AIF, albeit with an inherent systematic bias. Truncation of the dynamic PET scan duration to less than 100 min reduced the stability of the kinetic modelling outputs. Quantification of [^18^F]LW223 uptake kinetics in the brain and heart required the use of different outcome measures, with *BP*_*TC*_ more stable in the heart and *V*_*T*_ more stable in the brain.

**Conclusion:**

Modelling of [^18^F]LW223 PET showed the use of simplified IDIF is acceptable in the rat and the minimum scan duration for quantification of TSPO expression in rats using kinetic modelling with this radiotracer is 100 min. Carefully assessing kinetic outcome measures when conducting a systems level as oppose to single-organ centric analyses is crucial. This should be taken into account when assessing the emerging role of the TSPO heart-brain axis in the field of PET imaging.

**Supplementary Information:**

The online version contains supplementary material available at 10.1007/s00259-021-05482-1.

## Introduction

Quantification of the 18 kDa translocator protein (TSPO) expression in vivo using positron emission tomography (PET) imaging has proven challenging due to sub-optimal physicochemical properties of previously developed PET radiotracers targeting TSPO (e.g. high non-specific binding and short half-life [1]), complex binding kinetics attributed to the human rs6971 genetic polymorphism [2, 3], and ubiquitous expression of TSPO in mammalian tissues throughout the body [4–9]. Consequently, accurate quantification of TSPO PET imaging datasets has predominantly relied on invasive arterial blood collections to generate arterial plasma input function (AIF) data for kinetic modelling.

We have recently developed the TSPO PET radiotracer [^18^F]LW223, which has binding not susceptible to the human rs6971 genetic polymorphism and is able to detect and quantify macrophage-driven inflammation in a rat myocardial infarction (MI) model and explore the heart-brain axis that exists in this pathology [10]. This radiotracer has a distinct kinetic profile in brain versus heart, and therefore lends itself as an optimal compound for understanding the impact of different binding kinetics on outcome measures.

Although used as the “gold-standard” research method, invasive kinetic analyses are not feasible in routine large-scale clinical nuclear medicine studies. Furthermore, in small animal research, “gold-standard” invasive kinetic modelling is complicated by the limited blood volume of rodents, which requires specific set-ups such as automated blood sampling equipment and surgically induced arterial-venous shunts [11]. This also limits the possibility of performing longitudinal studies which aim to investigate disease progress. To circumvent the issues associated with using AIF for quantification of TSPO PET datasets, simplified methods of quantification have been proposed, including the use of image-derived input functions (IDIF) [12].

We and other colleagues have reported that increased TSPO in the heart due to myocardial infarction begets increased neuronal TSPO expression [5, 13]. The pathological impact of this heart-brain inflammatory axis is yet to be determined but its existence highlights the importance of seeing beyond single-organ centric analysis while pinpointing the need to characterise performance and stability of traditional kinetic models and kinetic constants. Thus far, use of these models and constants is more common in brain PET research, and their application to multiple organs with varying degrees of target expression and radiotracer kinetics has not been as widely explored compared with the use of simplified outcome measures such as SUV.

This paper aims to provide a comprehensive statistical assessment of ^18^F-LW223 heart and brain kinetic measures when using “gold-standard” AIF versus simplified IDIF analysis protocols of dynamic PET datasets. This is an important component of radiotracer validation, and will also provide a better understanding of some of the complexities associated with analysing the emerging heart-brain axis in the field of TSPO PET imaging.

## Materials and methods

### Animals and surgical procedures

The data collection and original analysis was described in our previous manuscript [10]. All experiments were authorised by the local University of Edinburgh animal welfare and ethical review committee and in accordance with the Home Office Animals (Scientific Procedures) Act 1986. Fifteen adult male Sprague–Dawley rats (251 ± 4 g) were used. The animals were housed under standard 12 h light:12 h dark conditions with food and water available ad libitum. For MI surgeries, anaesthesia was induced and maintained using isoflurane (0.5–3% in 1.0 l/min oxygen) before buprenorphine (0.05 mg/kg, Alstoe Ltd, York, UK) was administered preoperatively for analgesia. Tracheal intubation was achieved under direct vision, and ventilation was maintained with a rodent ventilator (Harvard Apparatus Model 683, MA, USA, tidal volume 2.5 cm^3^, respiratory rate 60/min). MI was induced as previously described [14] and MI rats were imaged on 7 ± 1 days post-MI. The naive rat cohort had no surgical procedures and were imaged at a similar timepoint to the MI cohort.

### PET/CT imaging

Anaesthesia was induced and maintained with 1.5–2.5% isoflurane (50/50 oxygen/nitrous oxide, 1 l/min). An intravenous (i.v.) line was established in the tail vein for injection of the radiotracer and the femoral artery and vein were cannulated to allow automated blood sample collection, as previously described [11]. The whole blood arterial input function measured by the automatic blood sampler (Swisstrace GmbH, Switzerland) was corrected for the plasma-to-whole blood ratio and for metabolism in vivo (population based), as detailed in our previous manuscript [10]. The average inter-rat variability for the plasma-to-whole blood ratio was 29.3% and for the metabolism ranged between 2.7% (2 min) to 30.9% (60 min). Simulations were carried out on the impact of this level of variability on modelling outputs which demonstrated, unsurprisingly, that a quantitative bias is present (Supplementary Fig. 1). Body temperature was maintained by heated scanner bed or heated mat and monitored by rectal thermometer. Vital signs, including heart rate and respiration rate were monitored continuously during the experiments. PET/CT scans were acquired alongside i.v. bolus injection of [^18^F]LW223 (23.1 ± 1.7 MBq).

Data were acquired using a PET/CT small animal scanner (nanoPET/CT, Mediso, Hungary). A CT scan (semi-circular full trajectory, maximum field of view, 480 projections, 50 kVp, 300 ms and 1:4 binning) was acquired for attenuation correction. A 120-min emission scan was obtained using 3-dimentional 1:5 mode and re-binned as follows: 18 × 10 s; 2 × 30 s; 1 × 60 s; 2 × 2 min; 10 × 5 min; 6 × 10 min. PET studies were reconstructed using Mediso’s iterative Tera-Tomo 3D reconstruction algorithm, which includes point spread function correction, and the following settings: 4 iterations, 6 subsets, full detector model, low regularisation, spike filter on, voxel size 0.4 mm and 400–600 keV energy window. PET data were corrected for randoms, scatter and attenuation. Ten seconds was the shortest framing possible in this study as the TeraTomo reconstruction algorithm failed to quantitatively reconstruct low count frames less than this.

### Image processing and analysis

Reconstructed scans were imported into PMOD version 3.8 (PMOD Technologies, Switzerland). Volumes of interest (VOIs) were manually drawn around the heart and brain using CT images. To sample the infarct area (or equivalent area in naive hearts) averaged PET images (0–120 min) were used to place three spherical VOIs at the centre of the infarct territory within the ventricular wall (1.5 mm^3^). The IDIF was derived from a VOI placed in the left ventricle of the heart (7 voxel size with each voxel = 0.4 × 0.4 × 0.4 mm) rather than the whole chamber to minimise spill-over influence from the ventricle wall. Time activity curves were generated and standard uptake values (SUVs) calculated as concentration in the VOI divided by injected dose divided by animal weight. Kinetic modelling was performed using the two-tissue compartment model (2TCM) with *v*_*B*_ constrained to 0.05 to estimate the kinetic rate constants *K*_*1*_ to *k*_*4*_ and *V*_*T*_ in different tissues. 2TCM with time delay corrections of invasive blood data was the most suitable model for the data in these organs based on our previous work, where the full kinetic outcome parameters for the regions used in this study can also be found [13]. The choice of modelling approach and constraints was based on the best compromise between goodness of fitting (AIC) and robustness of fitting (%SE and outcome measure stability). The binding potential relative to non-displaceable volume (*BP*_*ND*_) was defined as *k*_*3*_/*k*_*4*_ [15]. The transfer corrected *BP*_*ND*_, termed *BP*_*TC*_, was formulated as previously published [13] and as detailed below:

$${BP}_{TC}=\frac{k_3}{k_4}\div K_1$$

where *k*_*3*_ = radiotracer association rate with specific binding, *k*_*4*_ = dissociation rate constant of target-ligand and *K*_*1*_ = rate constant for transfer from arterial plasma to tissues.

For investigation of the impact of dynamic PET timeframe truncation on modelling outcomes, the 120-min dataset was truncated by 20, 40, 60, 70, and 80 min.

### Statistical analysis

GraphPad Prism version 6 (GraphPad Software Inc., USA) was used for statistical analysis and production of graphs. Were appropriate, data are show as the mean ± standard error of the mean (SEM). Pearson correlations were used to determine the strength of associations, with a *p* < 0.05 considered statistically significant. To assess kinetic outcome measures’ stability, the intraclass correlation coefficient (ICC) was calculated as below:

$$ICC=\frac{\sigma_S^2}{\sigma_S^2+\sigma_e^2}$$

where ${{\sigma }}_{S}^{2}$ = true variance and ${{\sigma }}_{e}^{2}$ = error variance.

## Results

### PET outcome measures estimated using AIF and IDIF

**Fig. 1:**
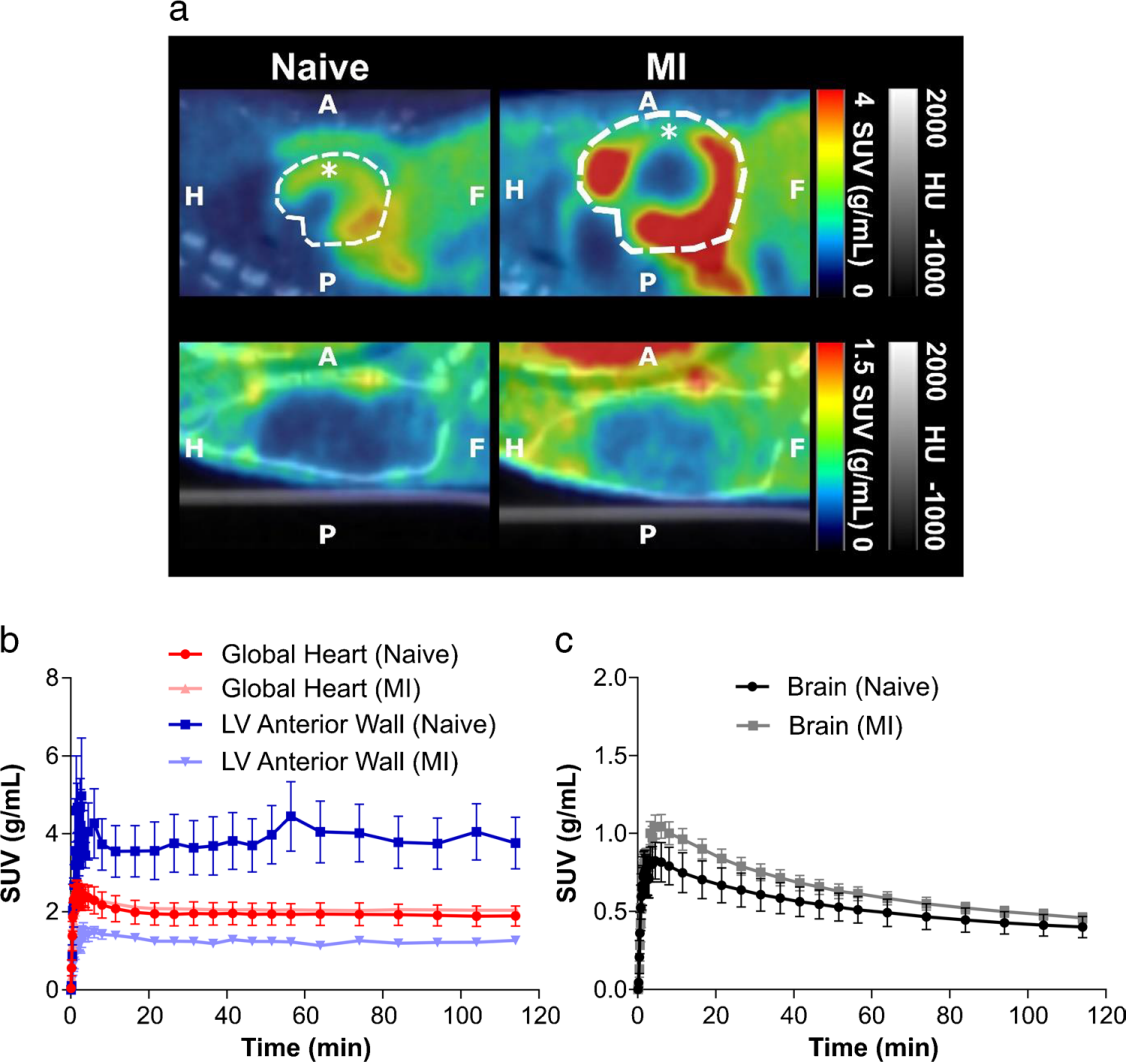
[^18^F]LW223 distribution and kinetics in naive rats and rats with myocardial infarction (MI). **a** Example sagittal SUV images, which are non-perfusion corrected, top row shows the heart and the bottom row the brain. The cardiac areas typically included in the CT drawn global heart VOI are indicated by the broken white line, and the myocardium subsampled within the anterior wall is indicated by a single asterisk (*). A, anterior; P, posterior; H, head and F, foot. **b** SUV time activity curves for VOIs in the heart and **c)** brain. Mean ± SEM, *n* = 6 for naive and *n* = 9 for MI

Following MI, there is an upregulation of [^18^F]LW223 binding within the heart, although in absence of perfusion correction this results in a lower SUV within the infarct (LV anterior wall, Fig. 1a–b). The kinetics of [^18^F]LW223 in the healthy and infarcted heart display a Morrison’s kinetics binding profile, whereas in the brain the kinetics can be described by the Michaelis–Menten binding principles (Fig. 1b–c) [13].

**Fig. 2:**
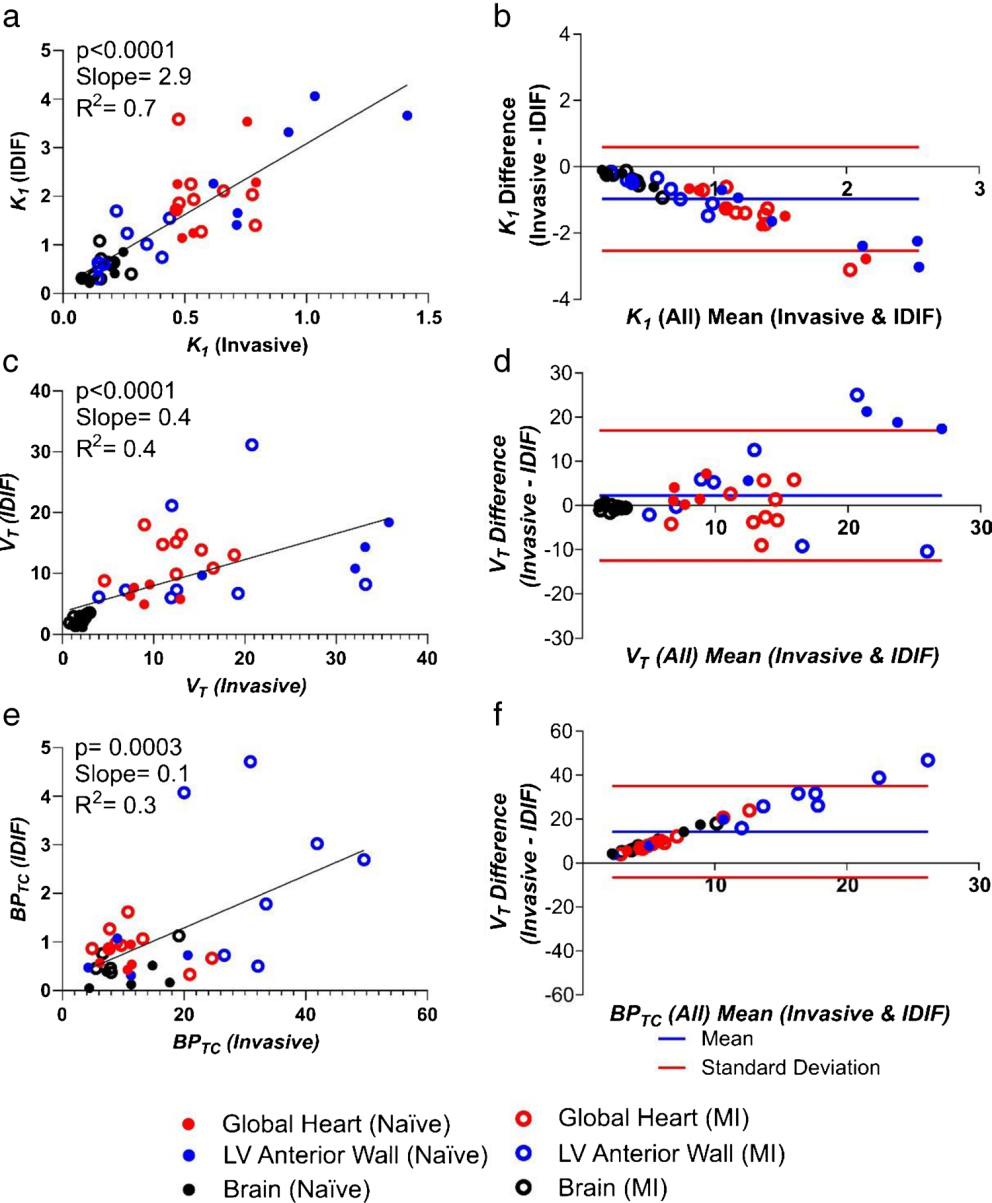
Comparison of PET outcome measures calculated using the “gold standard” invasive AIF and IDIF in all rats. **a** Correlation of *K*_*1*_ calculated using AIF vs*.* IDIF and **b** Bland–Altman plot for the same comparison. **c** Correlation of *V*_*T*_ calculated using AIF vs. IDIF and **d** Bland–Altman plot for the same comparison. **e** Correlation of *BP*_*TC*_ calculated using AIF vs*.* IDIF and **f** Bland–Altman plot for the same comparison. *n* = 15 for all graphs (6 naive animals and 9 MI animals) with 3 regions per animal (heart, brain and left ventricular anterior wall)

**Fig. 3:**
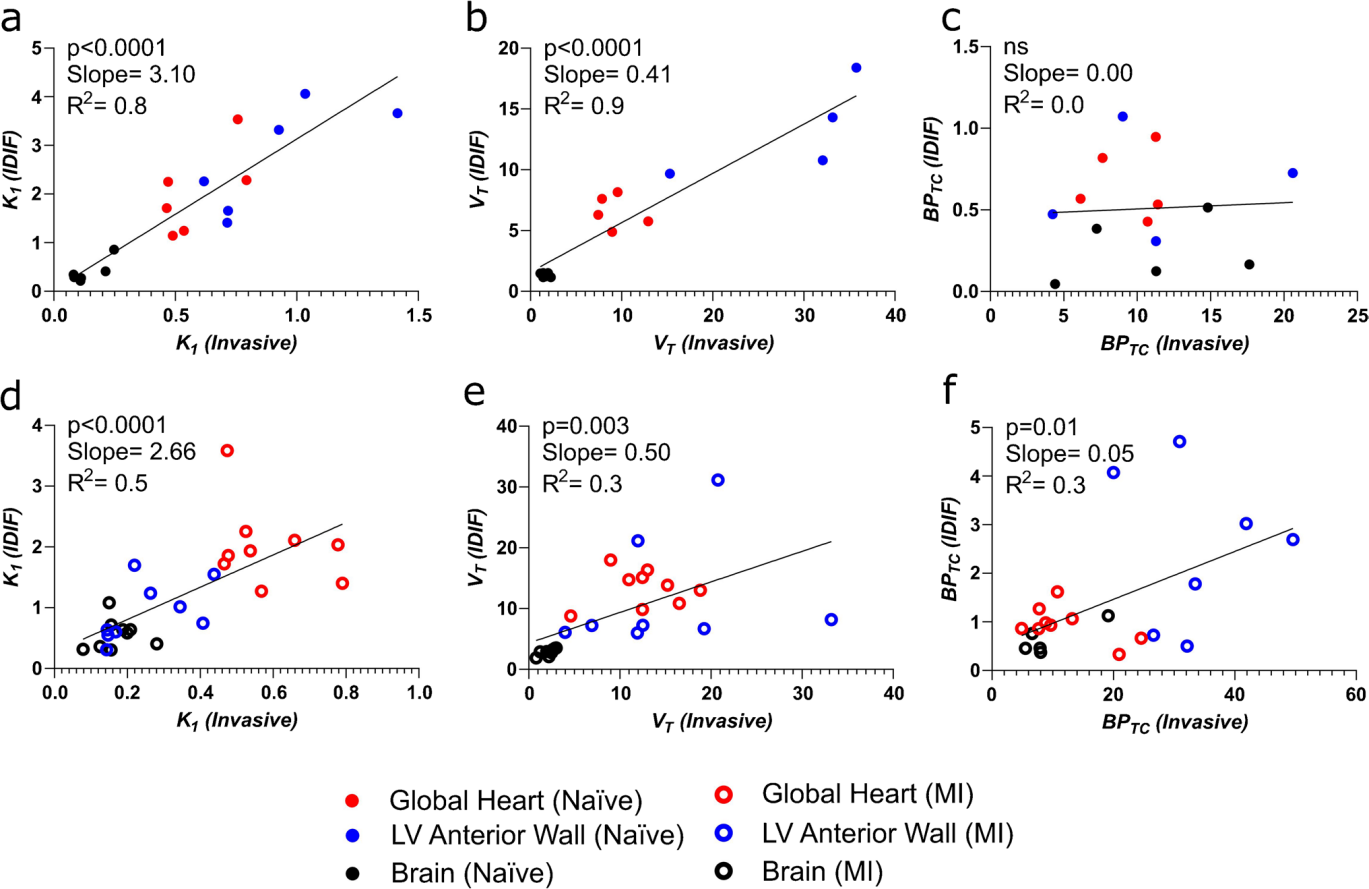
Comparison of PET outcome measures calculated using invasive AIF and IDIF in separate naive and MI cohorts**. a** Correlation of *K*_*1*_, **b** *V*_*T*_ and **c** *BP*_*TC*_ calculated using AIF vs. IDIF in naive cohort heart and brain. *n* = 6 animals with 3 regions (heart, brain and left ventricular anterior wall). **d** Correlation of *K*_*1*_, **e** *V*_*T*_ and **f** *BP*_*TC*_ calculated using AIF vs. IDIF in the MI cohort heart and brain. *n* = 9 animals with 3 regions (heart, brain and left ventricular anterior wall)

The average whole blood time activity curves for AIF and IDIF are shown in Supplementary Fig. 2. Use of IDIF for 2TCM modelling generates outcomes which correlate with those generated using invasive AIF (Fig. 2). Overall, compared to using the invasive AIF, *K*_*1*_ values were 190% higher when using IDIF (based on slope, Fig. 2a–b). The other 2TCM microparameters were higher (*k*_*4*_), lower (*k*_*3*_) and same (*k*_*2*_) when using IDIF (Supplementary Fig. 3). *V*_*T*_ and *BP*_*TC*_ were 60% (based on slope, Fig. 2c and d) and 90% lower respectively (based on slope, Fig. 2e and f) when using IDIF compared to AIF. This systematic bias when using IDIF is evident within the Bland–Altman plots (Fig. 2b, d and f). When analysing the comparison between AIF and IDIF in naive and MI cohorts on their own, a similar pattern is evident although the fitting is generally poorer within those with MI (Fig. 3).

### The impact of truncating PET scan duration on kinetic model outputs

**Fig. 4:**
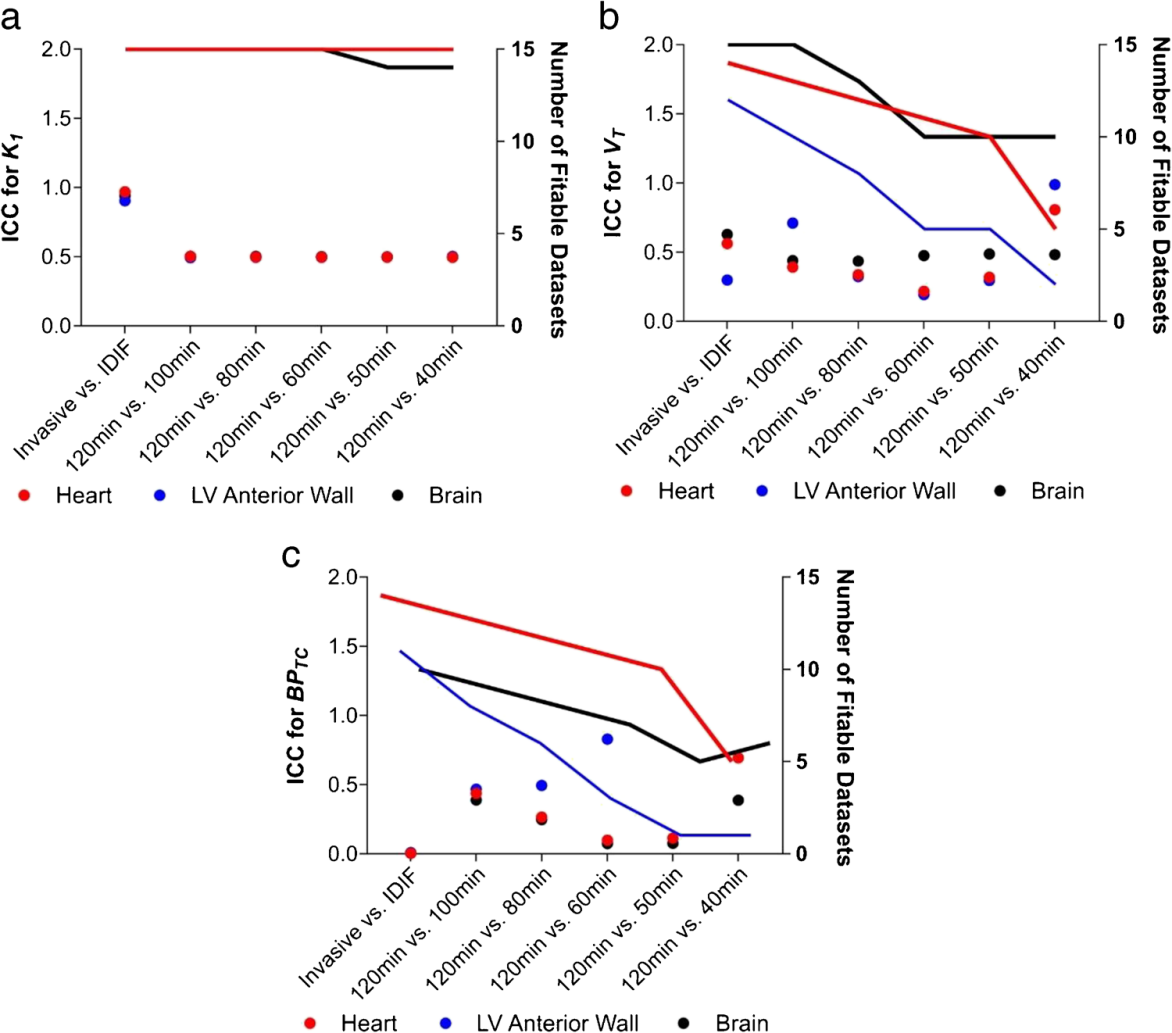
The ICC of 2TCM parameters for invasive AIF function, IDIF and PET frame truncation in all datasets. **a** The ICC for *K*_*1*_ calculated using the different conditions in naive and MI rats is shown as dots (left *Y* axis), with the lines detailing the number of datasets (rats) where calculation of *K*_*1*_ was possible (right *Y* axis). **b** The same analysis is shown for *V*_*T*_ and **c)** *BP*_*TC*_. *n* = 15 (6 naive animals and 9 MI animals)

The intraclass correlation coefficients (ICC) of *K*_*1*_, *V*_*T*_ and *BP*_*TC*_ were calculated for each truncation of the 120-min dynamic PET duration (Fig. 4). *K*_*1*_ ICC values were unaffected by truncations in scan duration (Fig. 4a). The *V*_*T*_ ICC demonstrates greater stability in brain outcomes for different truncations compared with heart outcomes (Fig. 4b). The improved brain performance versus the heart maybe due to the decreasing effect of the apparent quasi-irreversible kinetics on 2TCM (Fig. 1). Conversely, for *BP*_*TC*_, a truncation greater than 20 min begins to reduce the number of fittable datasets (defined as the organ data for one animal) and parameter stability in the brain (Fig. 4b and c), but less so in the heart. The kinetic constant values for the hypoperfused left ventricular anterior wall were the most affected by truncation of PET scan duration with the highest number of non-fittable datasets. When assessing the ICC of truncated data within the naive and MI cohorts separately, *K*_*1*_, *V*_*T*_ and *BP*_*TC*_ ICC results are overall comparable (Supplementary Fig. 4). However, a truncation greater than 20 min still results in loss of fittable datasets.

**Fig. 5:**
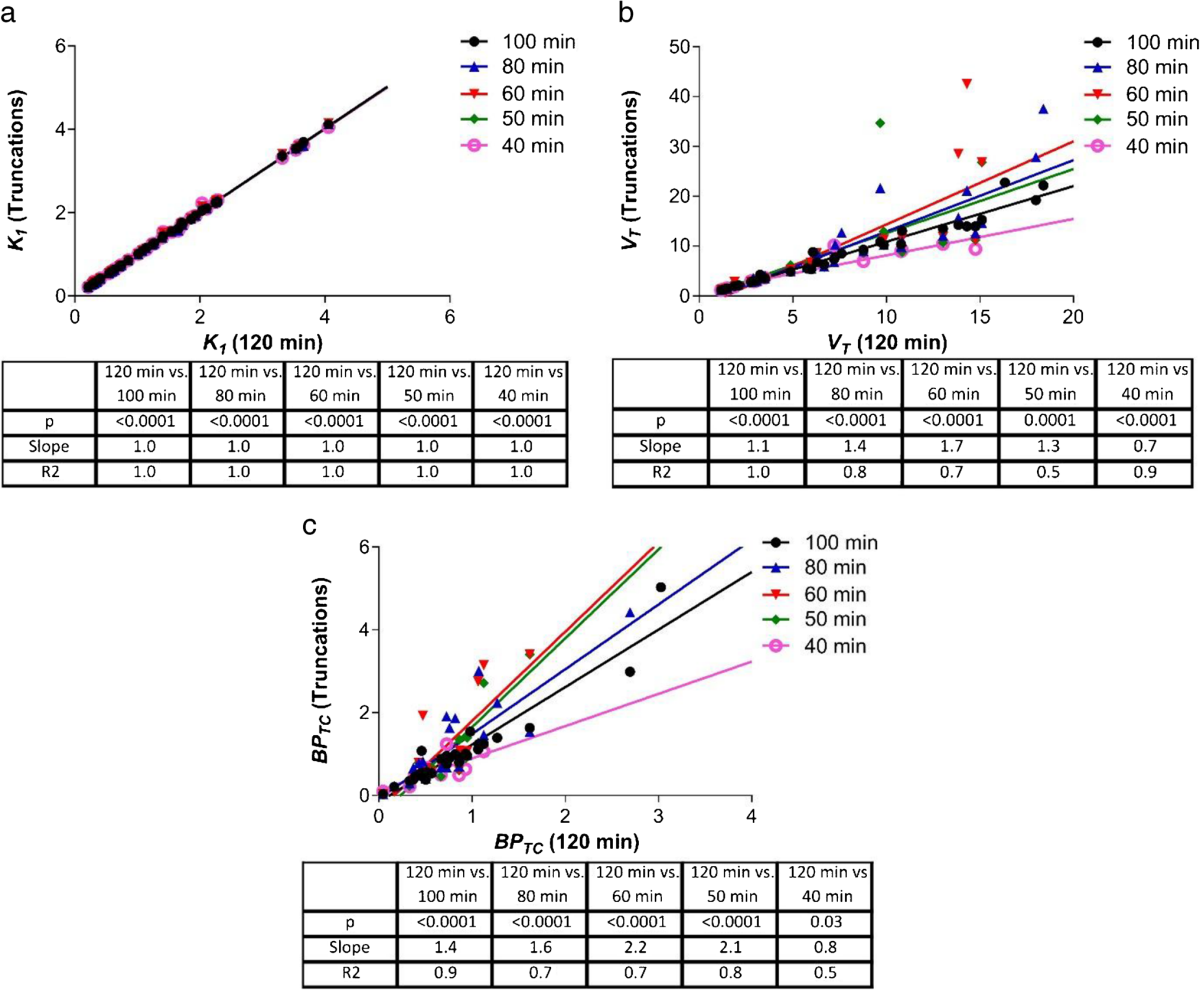
The impact of PET scan duration truncation on 2TCM parameter accuracy in all datasets. **a** Correlation between *K*_*1*_ calculated using a 120-min PET scan duration and 5 other truncated durations. **b** The same is shown for *BP*_*TC*_ and **c** *V*_*T*_. *n* = 15 (6 naive animals and 9 MI animals) with 3 regions per animal (heart, brain and left ventricular anterior wall)

Furthermore, a truncation of greater than 20 min impacts the quantitative accuracy of *V*_*T*_ and *BP*_*TC*_, but not *K*_*1*_, as is evidenced by the deteriorating *R*^*2*^ values and increasing measurement bias (regression line slope, Fig. 5). A similar pattern is seen when assessing the naive and MI cohorts separately (Supplementary Fig. 5, 6 & 7).

## Discussion

Here, we investigated bias and stability of [^18^F]LW223 outcome measures in PET heart and brain imaging from a statistical analysis point of view using “gold-standard” AIF and simplified IDIF analysis protocols. Our results showed that PET outcome measures estimated using IDIF correlate with those derived with invasive AIF, albeit with an inherent bias. This is in agreement with previous observations with other TSPO PET radiotracers [12] as well as other PET studies conducted in rats and mice [16]. This bias is likely due to spill-over issues with IDIF, which deteriorate over time due to radiotracer uptake in surrounding heart tissue. Furthermore, accurate description of a peak image–derived input function (fast perfusion phase) in rodents can be challenging due to the fast heart rate of these animals (c. 300–400 bpm) and limited count rate statistics [17]. VOI placement for IDIF generation is important and the vena cava can be a reliable and reproducible IDIF method for [^18^F]FDG kinetic modelling in mice, even without partial volume corrections [16, 18]. Albeit, useful in mice PET studies, in rat heart and brain PET experiments, the use of the left ventricle blood pool VOI is more amenable due to limited size of most preclinical PET scanners field of view and larger animal body size. Notwithstanding, because this bias is systematic and strongly correlated with the AIF results, it represents an acceptable trade-off between quantitative accuracy and simplification of protocols, facilitating technology adoption and aiding translational feasibility. Similarly to [^18^F]FDG metrics that show kinetic modelling provides the most quantitative outcomes [19], large-scale adoption of this radiotracer in the preclinical and clinical settings was pillared on the use of simplified outcome measures like SUV.

The Bland–Altman analysis is a graphical representation, whereby two biomedical techniques can be compared and can be used to assess agreement between two different methods. Baumgartner et al. suggested these plots should be used as a first step in the statistical analysis of radiotracer performance, as they can be useful to understand the dependence of variability on the measured signal and bias of measurements [20]. Our results showed that simplification of protocols (i.e., use of IDIF) resulted in an outcome measure quantitative bias but with a low number of outliers in the Bland–Altman plot; thus, indicating IDIF can produce reliable data.

The variability of PET outcome measures is dependent on organ-specific kinetics, where *V*_*T*_ outcomes derived using 120-min data, and AIF were more stable for the brain than the heart tissue. Michaelis–Menten formulations, which have been seamlessly applied to in vivo PET kinetic quantifications, are widely used to model reversible binding kinetic radiotracers [15]. However, in exceptional circumstances, reversibly binding kinetics may also be modelled by the Morrison formulations when there are violations of the free ligand approximation for ligands with high affinity combined with high density targets [21, 22]. TSPO expression in the heart is approximately 5 times higher than in the brain [13, 23, 24], and [^18^F]LW223 has sub- to nanomolar affinity for TSPO [13]. This results in a considerable fraction of the ligand rapidly binding to the target, thus depleting ligand pool before establishing equilibrium in the heart. In these kinetic circumstances, the macroparameter *V*_*T*_ is rate-limited by changes in radiotracer transfer into the tissue poll (*K*_*1*_). Importantly, our data showed that *K*_*1*_ and *BP*_*TC*_ had comparable relative variability for all tissues when using 120-min data and AIF, although the preferred outcome measure for the brain was *V*_*T*_ (higher ICC values versus *BP*_*TC*_).

According to Baumgartner et al., when describing properties of a given radiotracer, it is important to consider an overall measure across different VOIs and a region-specific measure of reliability in a priori tissue with confirmed biological relevance (e.g. myocardium following infarction). Their work showed that some VOIs had better statistical performance than others, which may be due to different uptake characteristics. Importantly, their study highlighted the utility of comparing statistical performance of the same VOI from different radiotracers as well as different VOIs for the same radiotracer [20]. The work by Baumgartner et al. focused on single-organ (i.e. brain) multi-region analysis and foreseeably these differences of uptake kinetics are likely greater when investigating distinct organs, such as the heart-brain axis. Our results with [^18^F]LW223 also highlight the importance of validating quantification methods per radiotracer and per organ. For example, the contrasting kinetics in the brain and heart measured with [^18^F]LW223 in the rat would discourage the use of brain and heart coupling techniques previously proposed to stabilise [^18^F]GE180 outcome measures in the mouse [25]. It is likely that the differences in organ kinetics with [^18^F]LW223 PET in rats will also be present in other species, and it should therefore be a factor to consider as research moves into different species towards human translation.

The size of a VOI can also influence noise properties in that region, which in turn can have an impact on variability metrics [20]. This can explain why the left ventricular anterior wall VOI performed worse than the whole heart and whole brain VOI when using 120 min of data and AIF. Although the left ventricular anterior wall VOI placement was conducted in a uniform fashion across studies, the regional territory infarcted following MI surgery may be different depending on affected vessels downstream of the artery ligation. Another limitation of this work is the requirement for a long anaesthesia period (100–120 min), with the need to closely monitor animals to minimise complications such as respiratory depression, particularly across longitudinal studies. Isoflurane may also have a cardioprotective action on the heart, and it has been shown to affect the uptake of other PET radiotracers in the past [26, 27]. In addition, sampling requirements for dynamic IDIF can make accurate description of peak input function challenging, owing to the fast heart rate in small animals and limited count rate statistics.

In conclusion, quantification of [^18^F]LW223 uptake kinetics in the brain and heart require the use of *V*_*T*_ for quantification of TSPO in the brain and *BP*_*TC*_ in the hypoperfused heart. This highlights the importance of carefully assessing kinetic outcome measures when conducting different systems level as oppose to single-organ centric analyses. Statistical analysis of [^18^F]LW223 outcome measures showed the use of simplified IDIF is acceptable and the minimum scan duration for quantification of TSPO expression in rats with this radiotracer and kinetic modelling approach is 100 min. Taken together, these protocol simplifications will enable longitudinal PET imaging studies with this radiotracer in the rat.

### Supplementary Information

Below is the link to the electronic supplementary material.

Supplementary file1 (1.52 MB)

## Data Availability

All data is available on request to the corresponding author. We are currently in the process of adding the data to the digital repository Edinburgh DataShare.
