# Correction to: Modelling [^18^F]LW223 PET data using simplified imaging protocols for quantification of TSPO expression in the rat heart and brain

**DOI:** 10.1007/s00259-024-06781-z

**Published:** 2024-06-17

**Authors:** Mark G. MacAskill, Catriona Wimberley, Timaeus E. F. Morgan, Carlos J. Alcaide‑Corral, David E. Newby, Christophe Lucatelli, Andrew Sutherland, Sally L. Pimlott, Adriana A. S. Tavares

**Affiliations:** 1 University/ BHF Centre for Cardiovascular Science, University of Edinburgh, Edinburgh, UK; 2 Edinburgh Imaging, University of Edinburgh, Edinburgh, UK; 3 Centre for Clinical Brain Sciences, University of Edinburgh, Edinburgh, UK; 4 School of Chemistry, University of Glasgow, Glasgow, UK; 5 West of Scotland PET Centre, NHS Greater Glasgow and Clyde, Glasgow, UK

**Correction to: European Journal of Nuclear Medicine and Molecular Imaging (2021) 49:137–145**

10.1007/s00259-021-05482-1

The authors regret that there are some errors in the published original article.

Listed below are the corrections.

*The sentence:*

Overall, compared to using the invasive AIF, *K*_*1*_ values were 40% higher when using IDIF (based on slope, Figure 2.a-b).

*Should have read as:*

Overall, compared to using the invasive AIF, *K*_*1*_ values were 190% higher when using IDIF (based on slope, Figure 2.a-b).

*The sentence:*

The other 2TCM microparameters were also higher when using IDIF (10%-200% range, Supplementary Figure 3).

*Should have read as:*

The other 2TCM microparameters were higher (*k*_*4*_), lower (*k*_*3*_) and same (*k*_*2*_) when using IDIF (Supplementary Figure 3).

*The sentence:*

*V*_*T*_ and *BP*_*TC*_ were 40% (based on slope, Figure 2. c & d) and 60% lower respectively (based on slope, Figure 2.e & f) when using IDIF compared to AIF.

*Should have read as:*

*V*_*T*_ and *BP*_*TC*_ were 60% (based on slope, Figure 2. c & d) and 90% lower respectively (based on slope, Figure 2.e & f) when using IDIF compared to AIF.

*The sentence:*

When analysing the comparison between AIF and IDIF in naive and MI cohorts on their own, a similar pattern is evident although the fitting is poorer within those with MI (Figure 3).

*Should have read as:*

When analysing the comparison between AIF and IDIF in naive and MI cohorts on their own, a similar pattern is evident although the fitting is generally poorer within those with MI (Figure 3).

*The sentence:*

A truncation greater than 20 min results in lower *K*_*1*_ ICC values, and therefore stability, with the global heart VOI particularly impacted (Figure 4.a).

*Should have read as:*

*K*_*1*_ ICC values were unaffected by truncations in scan duration (Figure 4.a).

*The sentence:*

The *V*_*T*_ ICC demonstrates stability in brain outcomes for different truncations and an improvement of heart outcomes (Figure 4.b.).

*Should read as:*

The *V*_*T*_ ICC demonstrates greater stability in brain outcomes for different truncations compared with heart outcomes (Figure 4.b.).

*The sentence:*

The improved brain performance improvement versus the heart maybe due to the decreasing effect of the apparent quasi-irreversible kinetics on 2TCM (Figure 1).

*Should read as:*

The improved brain performance versus the heart maybe due to the decreasing effect of the apparent quasi-irreversible kinetics on 2TCM (Figure 1).

*The sentence:*

When assessing the ICC of truncated data within the naive and MI cohorts separately, *K*_*1*_, *V*_*T*_ and *BP*_*TC*_ ICC results are overall higher in the diseased cohort (Supplementary Figure 4).

*Should read as:*

When assessing the ICC of truncated data within the naive and MI cohorts separately, *K*_*1*_, *V*_*T*_ and *BP*_*TC*_ ICC results are overall comparable (Supplementary Figure 4).

*The sentence:*

Furthermore, a truncation of greater than 20 min impacts the quantitative accuracy of the outcomes as is evidenced by the deteriorating *R*^*2*^ values and increasing measurement bias (regression line slope, Fig. 5).

*Should read as:*

Furthermore, a truncation of greater than 20 min impacts the quantitative accuracy of *V*_*T*_ and *BP*_*TC*_, but not *K*_*1*_, as is evidenced by the deteriorating *R*^*2*^ values and increasing measurement bias (regression line slope, Figure 5).

Some of the figures are also incorrect.

*Incorrect Figure 2:* 
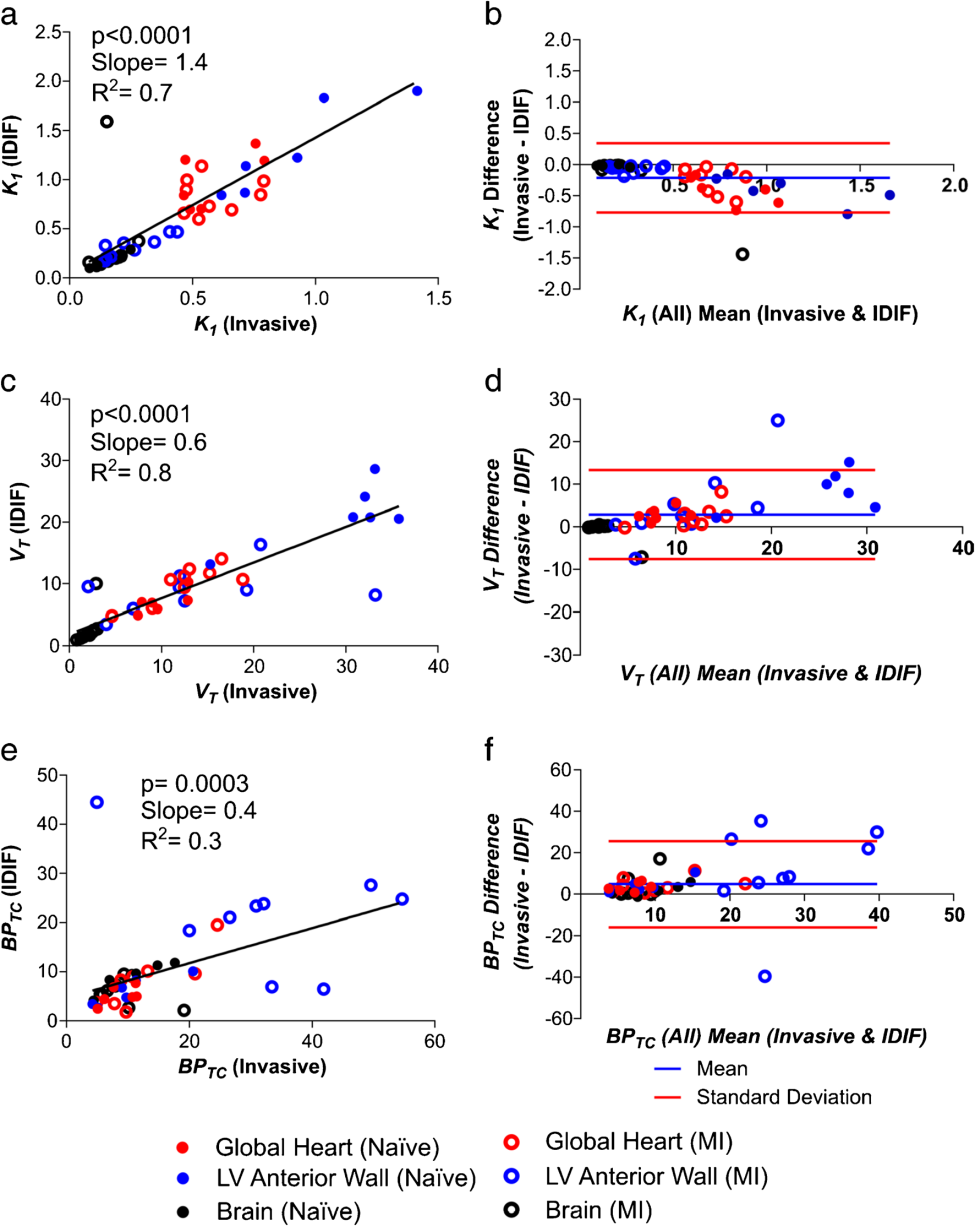


Figure 2. Comparison of PET outcome measures calculated using the “gold standard” invasive AIF and IDIF in all rats. a Correlation of *K*_*1*_ calculated using AIF vs. IDIF and b Bland–Altman plot for the same comparison. c Correlation of *V*_*T*_ calculated using AIF vs. IDIF and d Bland–Altman plot for the same comparison. e Correlation of *BP*_*TC*_ calculated using AIF vs. IDIF and f Bland–Altman plot for the same comparison. n = 15 for all graphs (6 naive animals and 9 MI animals) with 3 regions per animal (heart, brain and left ventricular anterior wall)

*Correct Figure 2:* 
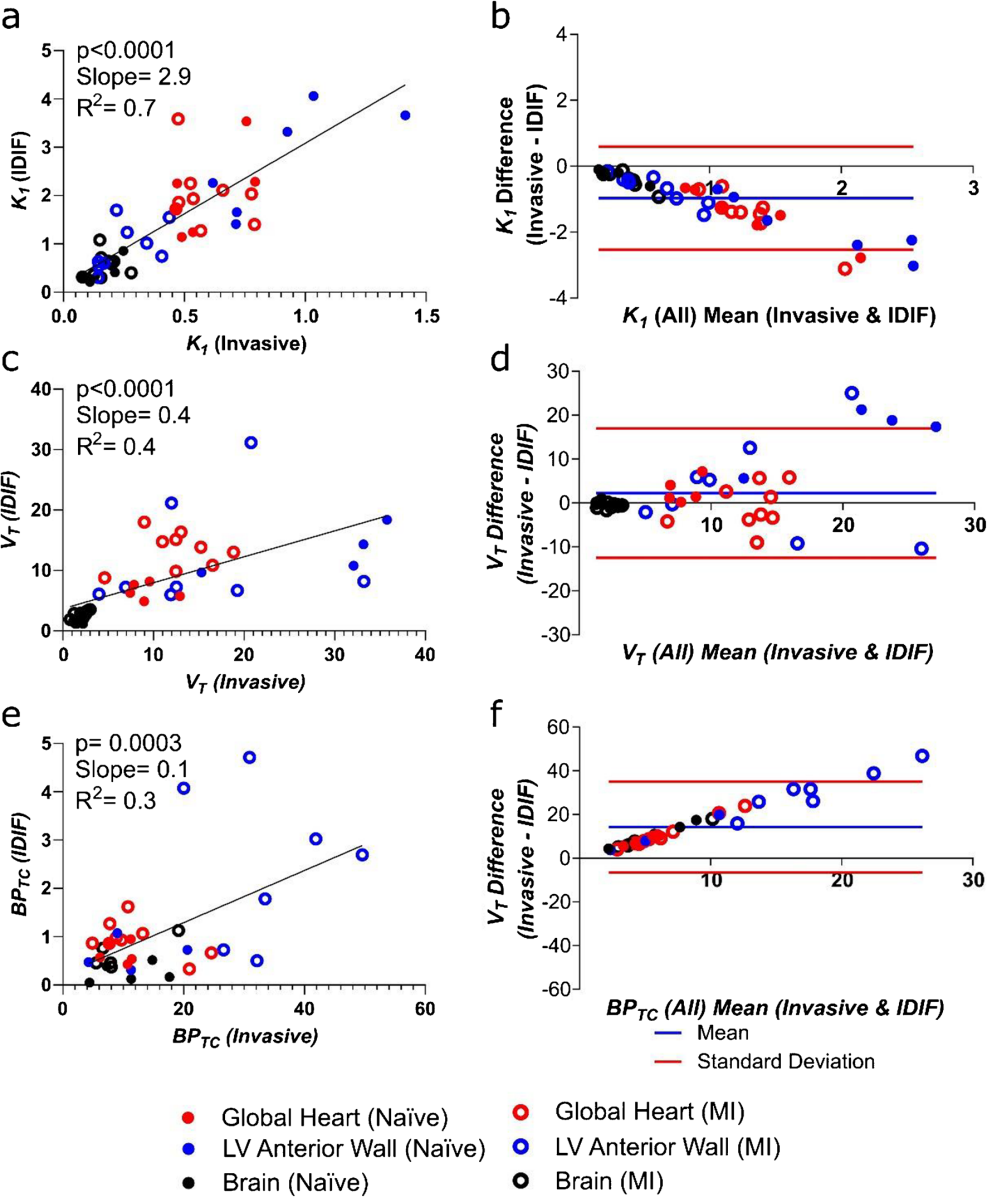


Figure 2. **Comparison of PET outcome measures calculated using the “gold standard” invasive AIF and IDIF in all rats. a)** Correlation of *K*_*1*_ calculated using AIF *vs.* IDIF and **b)** Bland–Altman plot for the same comparison. **c)** Correlation of *V*_*T*_ calculated using AIF *vs.* IDIF and **d)** Bland–Altman plot for the same comparison. **e)** Correlation of *BP*_*TC*_ calculated using AIF *vs.* IDIF and **f)** Bland–Altman plot for the same comparison. n=15 for all graphs (6 naive animals and 9 MI animals) with 3 regions per animal (heart, brain and left ventricular anterior wall).

*Incorrect Figure 3:* 
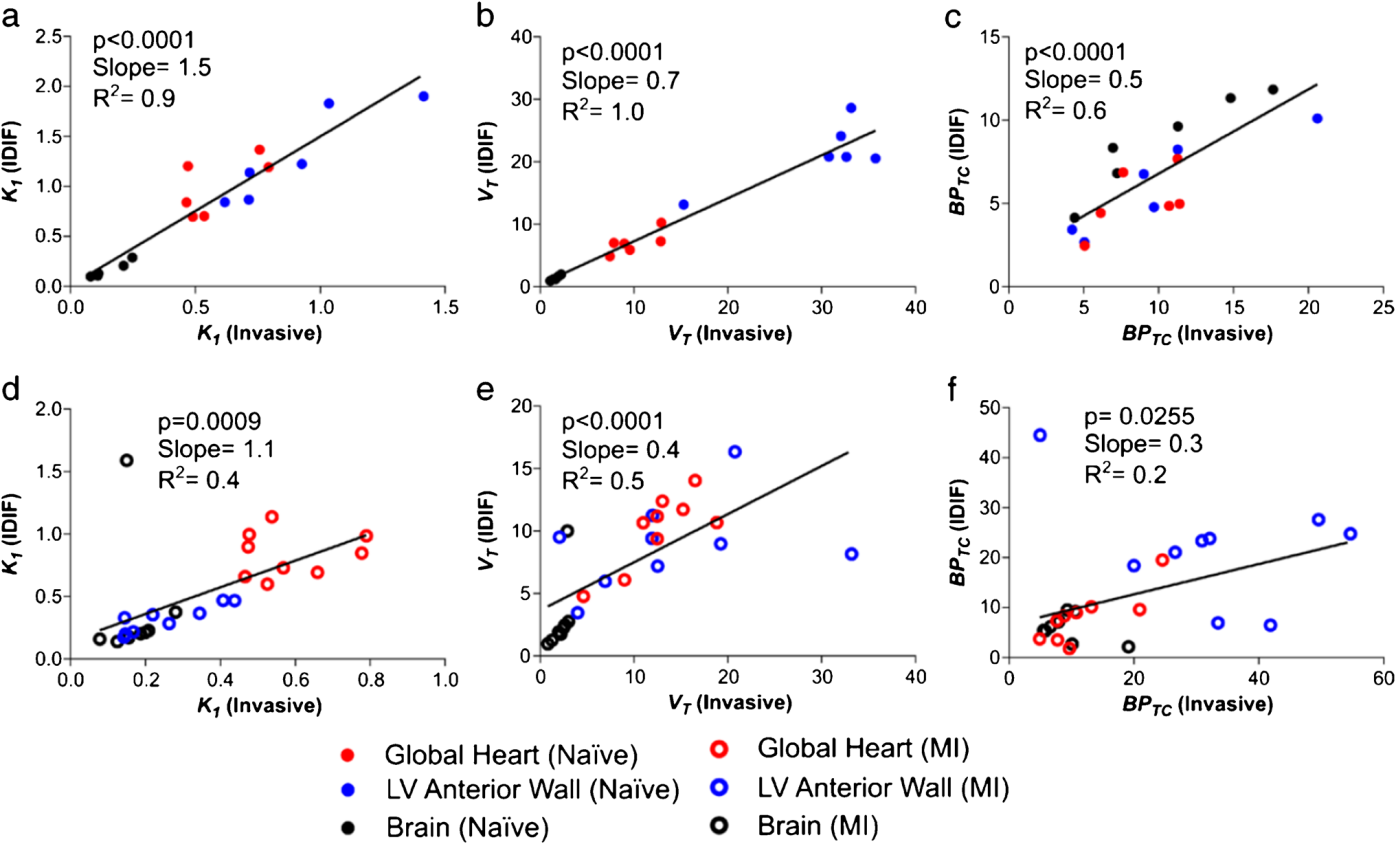


Figure 3. Comparison of PET outcome measures calculated using invasive AIF and IDIF in separate naive and MI cohorts. a Correlation of *K*_*1*_, b *V*_*T*_ and c *BP*_*TC*_ calculated using AIF vs. IDIF in naive cohort heart and brain. n = 6 animals with 3 regions (heart, brain and left ventricular anterior wall). d Correlation of *K*_*1*_, e *V*_*T*_ and f *BP*_*TC*_ calculated using AIF vs. IDIF in the MI cohort heart and brain. n = 9 animals with 3 regions (heart, brain and left ventricular anterior wall)

*Correct Figure 3:* 
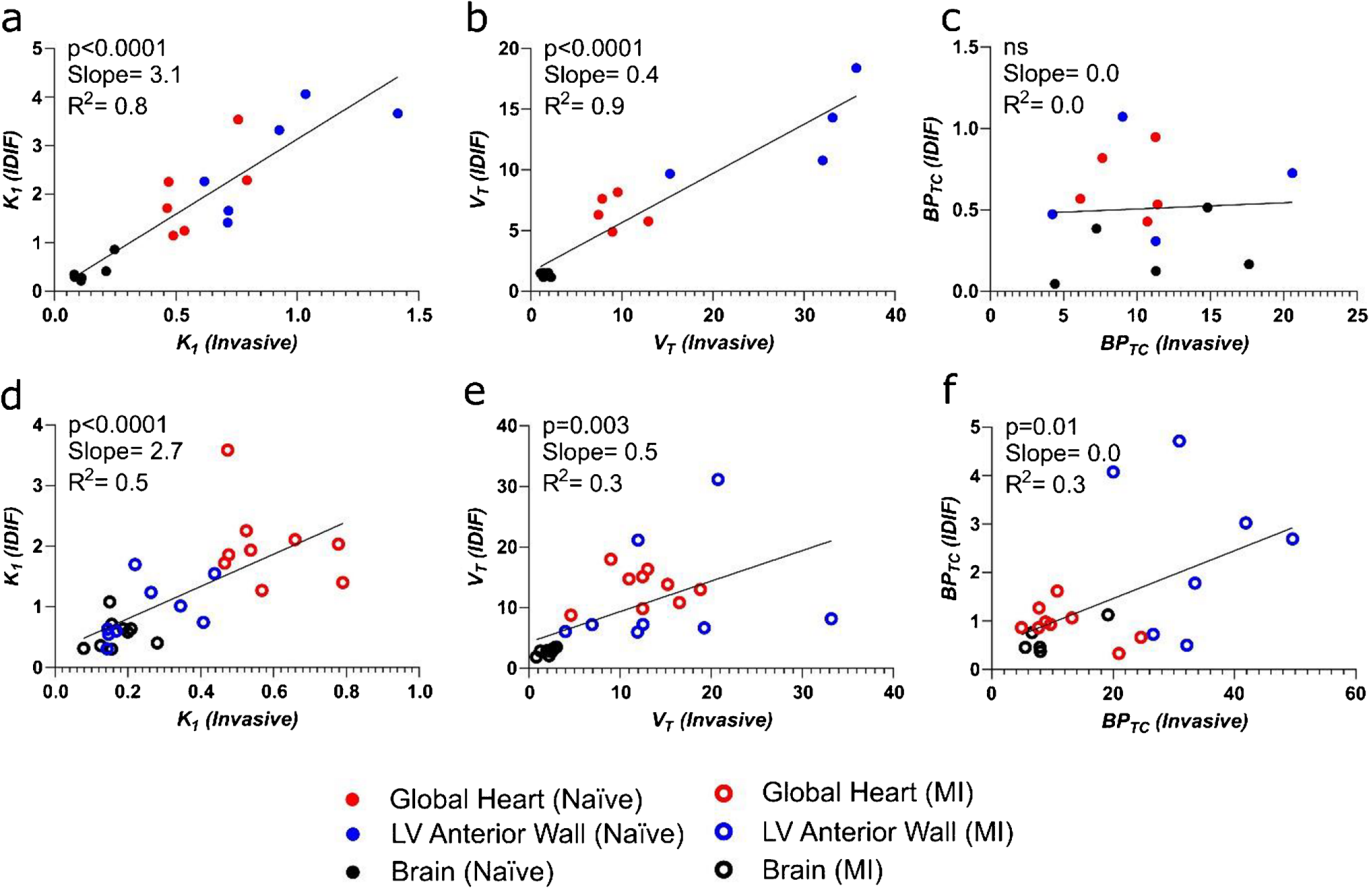


**Figure 3. Comparison of PET outcome measures calculated using invasive AIF and IDIF in separate naive and MI cohorts. a)** Correlation of *K*_*1*_, **b)** *V*_*T*_ and **c)** *BP*_*TC*_ calculated using AIF *vs.* IDIF in naive cohort heart and brain. n=6 animals with 3 regions (heart, brain and left ventricular anterior wall). **d)** Correlation of *K*_*1*_, **e)** *V*_*T*_ and **f)** *BP*_*TC*_ calculated using AIF vs. IDIF in the MI cohort heart and brain. n=9 animals with 3 regions (heart, brain and left ventricular anterior wall).

*Incorrect Figure 4:* 
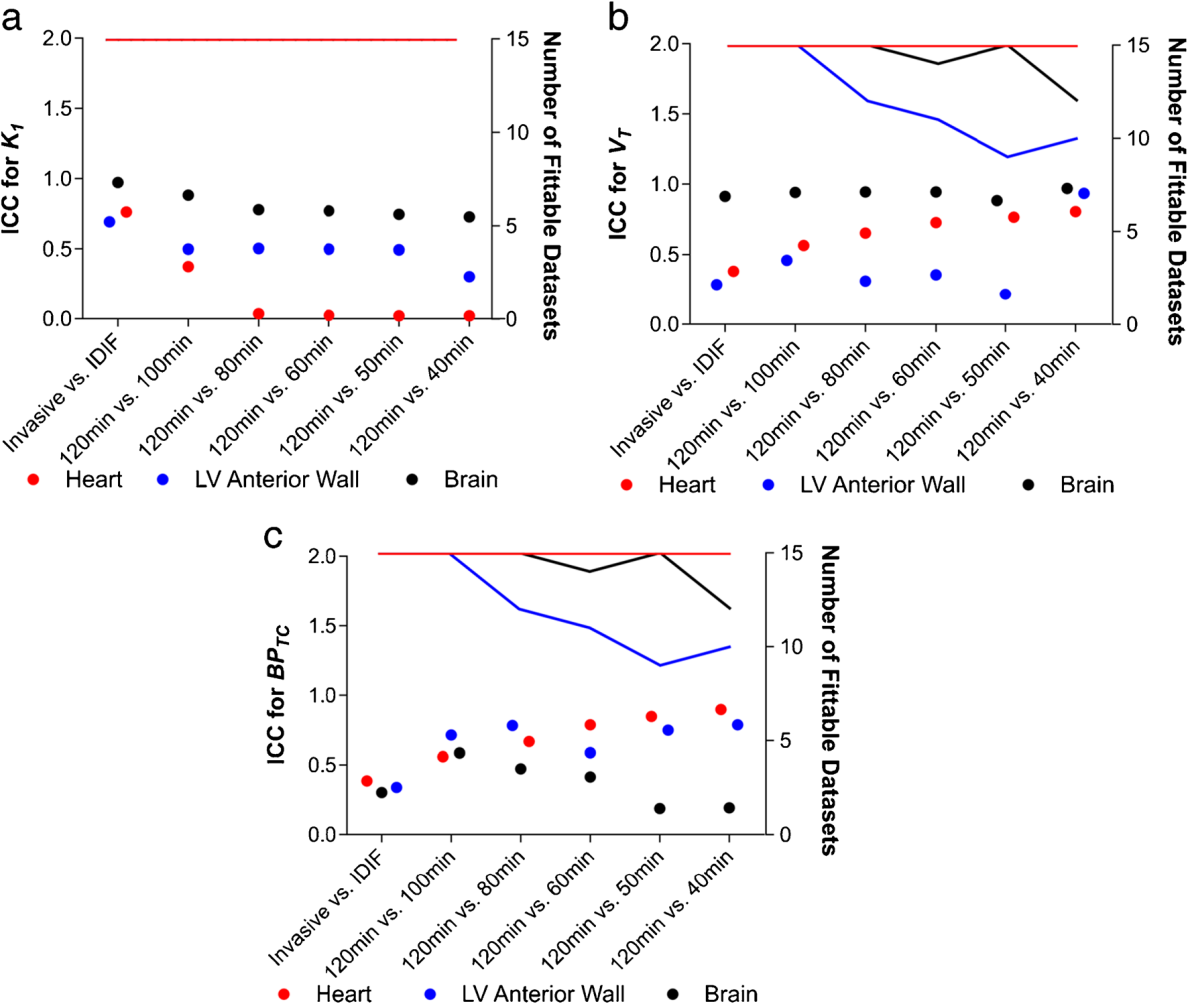


Figure 4. The ICC of 2TCM parameters for invasive AIF function, IDIF and PET frame truncation in all datasets. a The ICC for *K*_*1*_ calculated using the different conditions in naive and MI rats is shown as dots (left Y axis), with the lines detailing the number of datasets (rats) where calculation of *K*_*1*_ was possible (right Y axis). b The same analysis is shown for *V*_*T*_ and c) *BP*_*TC*_. n = 15 (6 naive animals and 9 MI animals)

*Correct Figure 4:* 
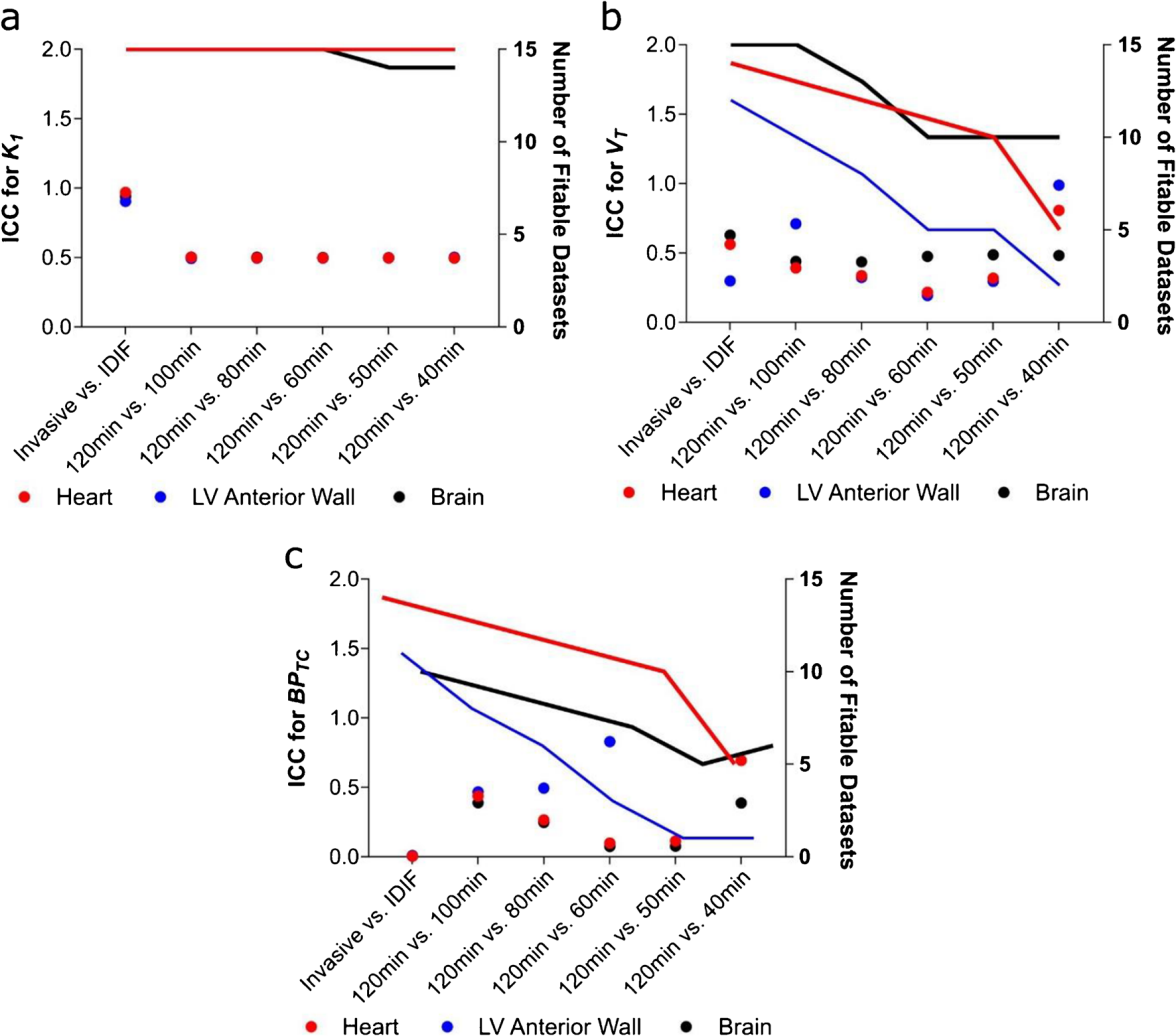


**Figure 4. The ICC of 2TCM parameters for invasive AIF function, IDIF and PET frame truncation in all datasets. a)** The ICC for *K*_*1*_ calculated using the different conditions in naive and MI rats is shown as dots (left Y axis), with the lines detailing the number of datasets (rats) where calculation of *K*_*1*_ was possible (right Y axis). **b)** The same analysis is shown for *V*_*T*_ and **c)** *BP*_*TC*_. n=15 (6 naive animals and 9 MI animals).

*Incorrect Figure 5:* 
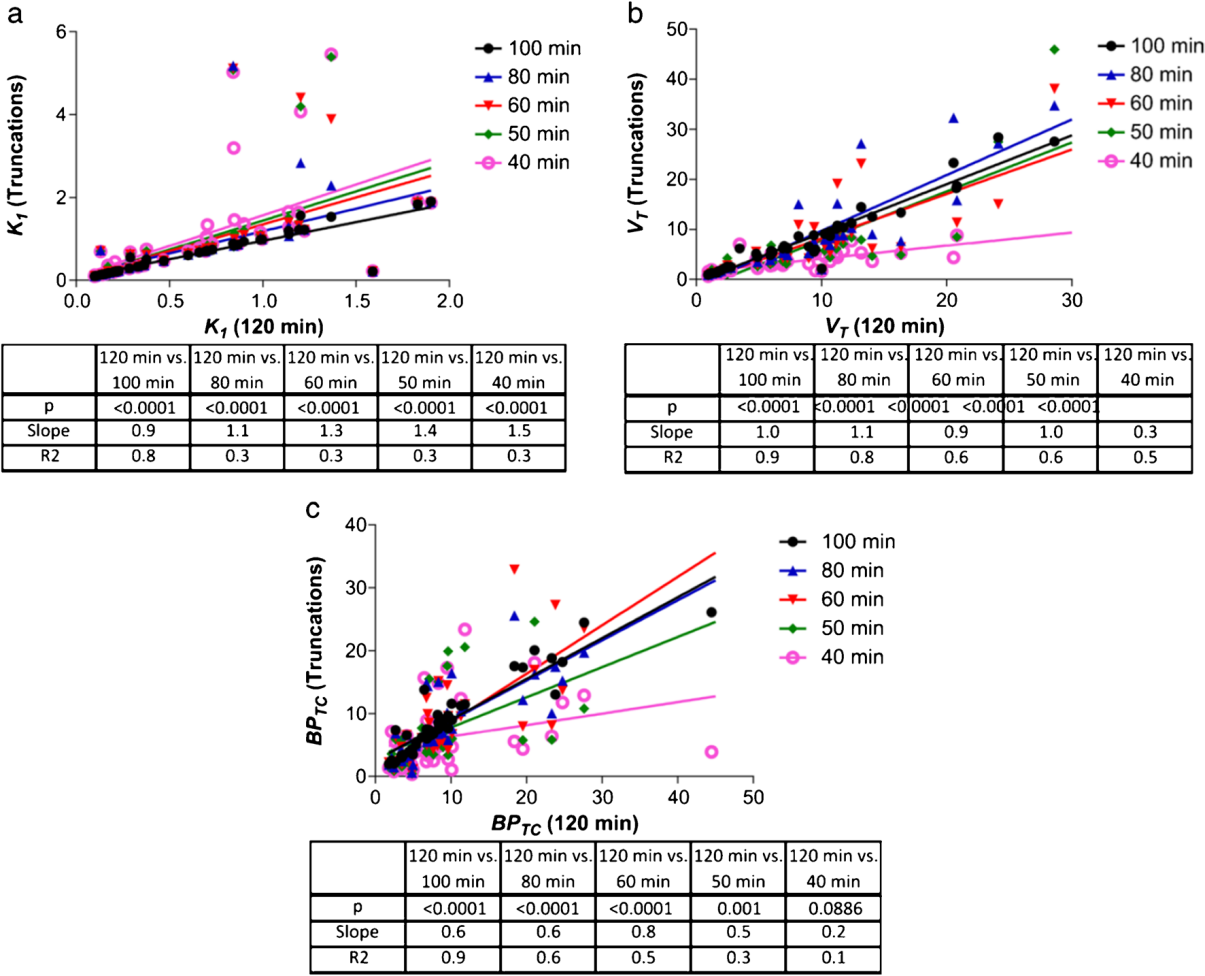


**Figure 5**. The impact of PET scan duration truncation on 2TCM parameter accuracy in all datasets. a Correlation between *K*_*1*_ calculated using a 120-min PET scan duration and 5 other truncated durations. b The same is shown for *BP*_*TC*_ and c *V*_*T*_. n = 15 (6 naive animals and 9 MI animals) with 3 regions per animal (heart, brain and left ventricular anterior wall)

*Correct Figure 5:* 
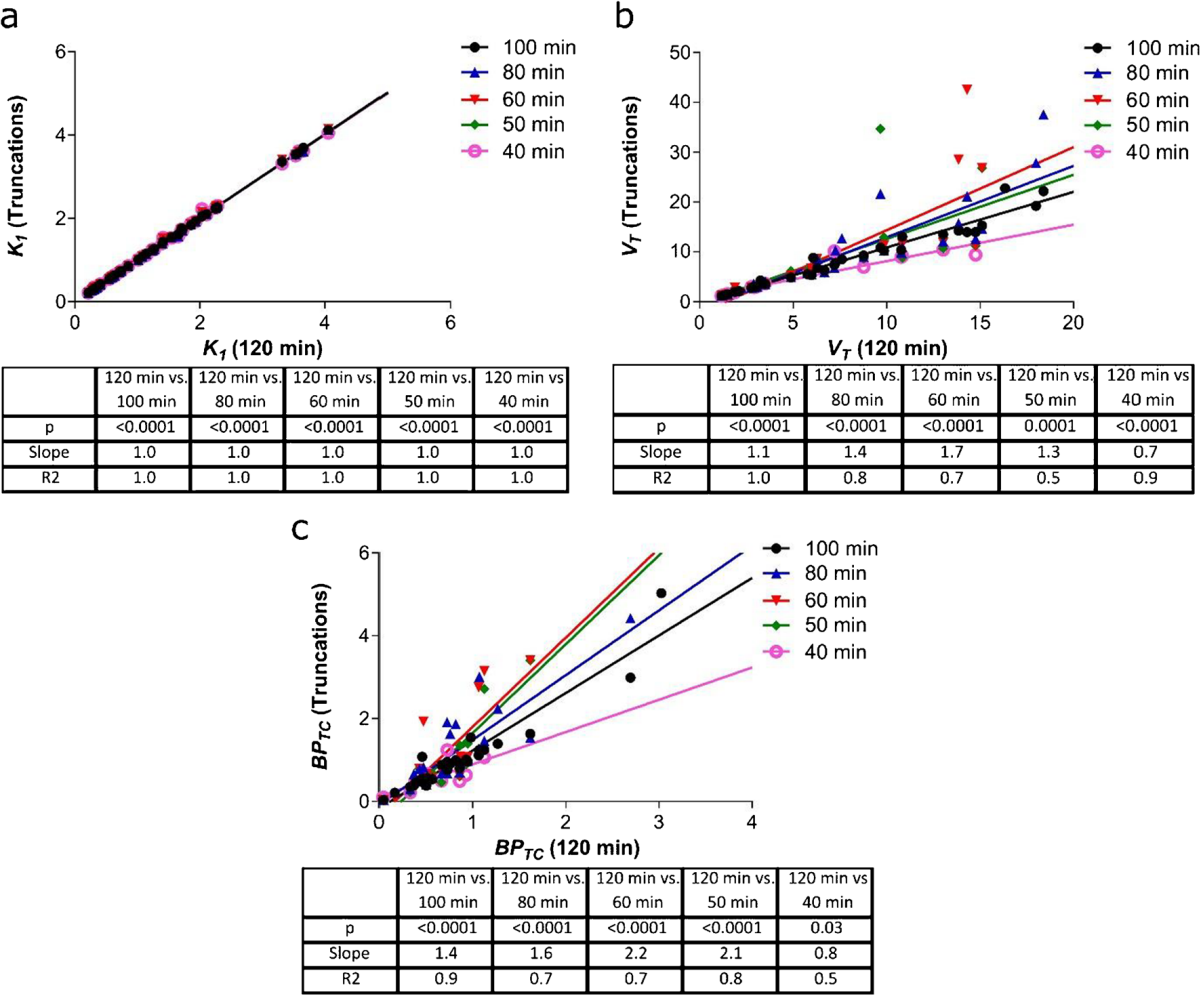


**Figure 5. The impact of PET scan duration truncation on 2TCM parameter accuracy in all datasets. a)** Correlation between *K*_*1*_ calculated using a 120 min PET scan duration and 5 other truncated durations. **b)** The same is shown for *BP*_*TC*_ and **c)** *V*_*T*_. n=15 (6 naive animals and 9 MI animals) with 3 regions per animal (heart, brain and left ventricular anterior wall).

The original article has been corrected.

