# Supplementary material for: Modelling [18F]LW223 PET data using simplified imaging protocols for quantification of TSPO expression in the rat heart and brain
Source: Eur J Nucl Med Mol Imaging. 2021 Aug 2;49(1):137–45. doi: 10.1007/s00259-021-05482-1 (PMC8712302; doi:10.1007/s00259-021-05482-1)
Supplement: Supplementary file 1 — Supplementary file1 (1.52 MB) [file 259_2021_5482_MOESM1_ESM.docx]

**Supplemental**

**Modelling [^18^F]LW223 PET data using simplified imaging protocols for quantification of TSPO expression in the rat heart and brain**

Mark G. MacAskill^1,2^, Catriona Wimberley^2,3^, Timaeus E.F. Morgan^1,2^, Carlos J. Alcaide-Corral^1,2^, David E. Newby^1^, Christophe Lucatelli^2^, Andrew Sutherland^4^, Sally L. Pimlott^5^, Adriana A.S. Tavares^1,2*^.

**Affiliations:**

1.University/ BHF Centre for Cardiovascular Science, University of Edinburgh, Edinburgh, UK.

2.Edinburgh Imaging, University of Edinburgh, Edinburgh, UK.

3.Centre for Clinical Brain Sciences, University of Edinburgh, UK.

4.School of Chemistry, University of Glasgow, UK.

5.West of Scotland PET Centre, NHS Greater Glasgow and Clyde, UK.

* Corresponding Author: [Adriana.Tavares@ed.ac.uk](mailto:Adriana.Tavares@ed.ac.uk)

**Supplemental Methods**

**Simulating the impact of variability in the radiometabolite curve on modelling parameters**

Two 'extreme' versions of a parent fraction curve were created by fitting the average parent fraction measurements with a 2 exponential and then multiplying the curve with a linear time series that varied between positive and negative 5% (at 2 minutes) and 27% (at 120 minutes), in line with the variability found at each time point of the measured parent fraction. The extracted IDIF was then corrected by the three parent fraction curves creating three corrected IDIF curves for input to a simulation model. Using the K1-k4 and vB values estimated in the previous paper by this group (Macaskill et al. 2020), three sets of curves were generated for heart and brain in naive and MI rat using a two tissue compartment model code developed in house in Matlab. To test the impact of the extreme parent fraction curves on the parameter estimates, the IDIF with original parent fraction was used with the three sets of curves generated. The parameters were estimated using the Turku fitting codes (fitk4).

**Supplemental Figures**

**
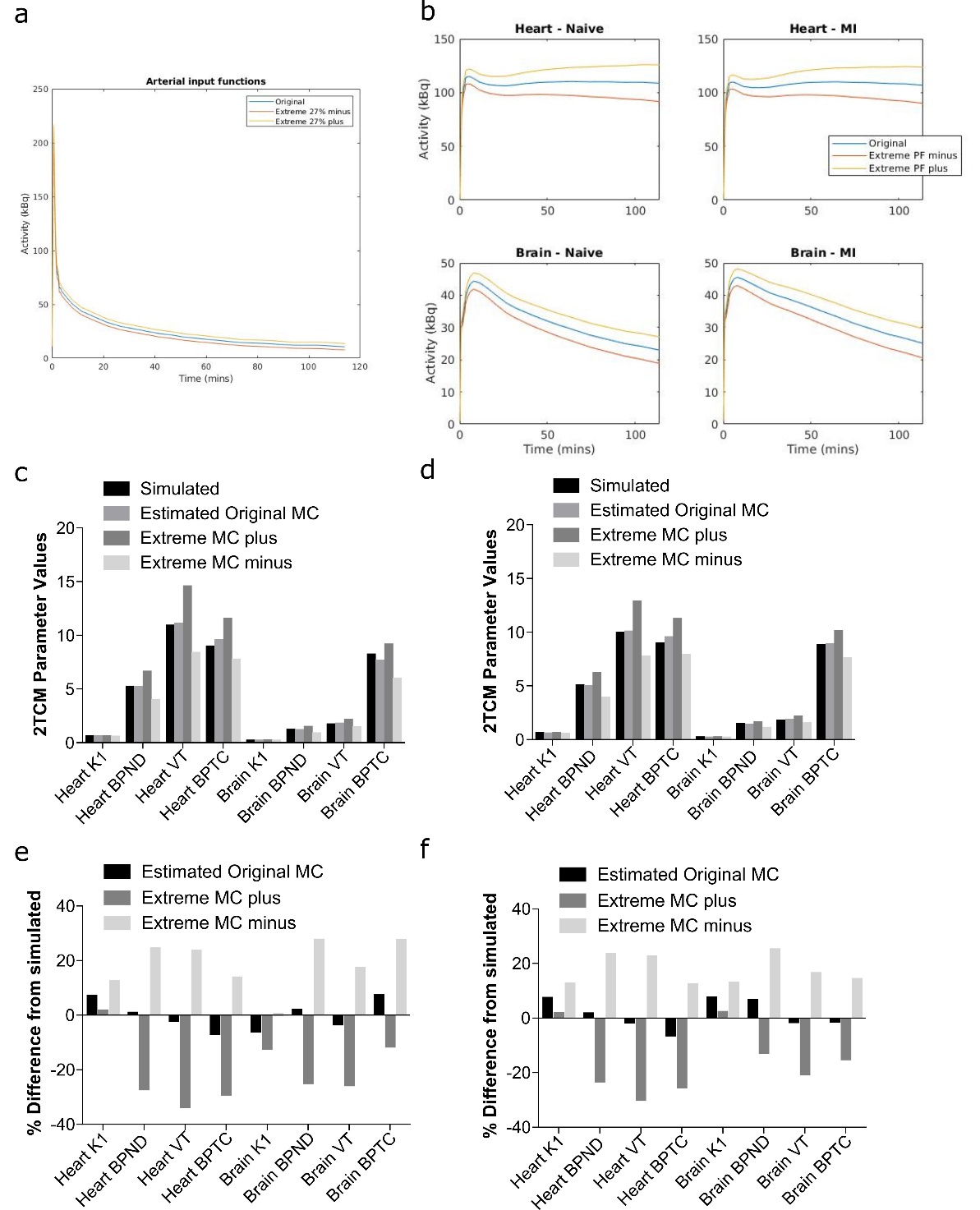
**

**Supplementary Figure 1. Simulation of the effect of radiometabolite curve variability on modelling parameters. a)** The three input functions used in the simulations which were the original parent curve correction and two examples of an extreme variation in the parent curve. **b)** The simulated TACs using the three different input curves for naive heart and brain as well as the MI heart and brain. The same input parameters (*K_1_-k_4_, vB*) are used for each set of three curves. **c)** The resultant parameter estimates for naïve and **d)** MI cohorts for each of the simulated set of curves. **e)** The % difference between estimated parameters and simulation input for naïve and **f)** MI cohorts.

**Supplementary Figure 2. Average whole blood time activity curves from arterial input function (AIF) and image-derived input function (IDIF).** The blood input curves for both approaches for all naïve and myocardial infarct (MI) rats were averaged together as shown. Graph represents mean ± SEM, n=15 (naïve n=6, MI n=9).**”**


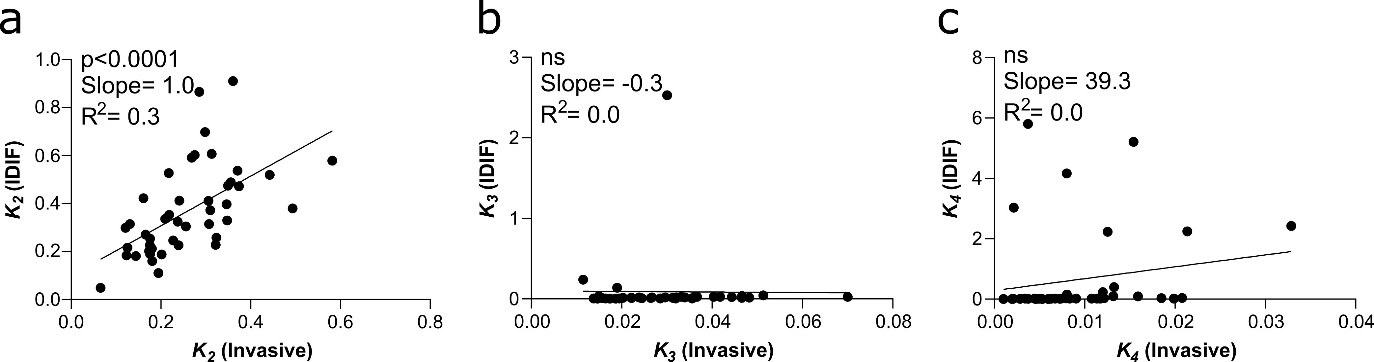


**Supplementary Figure 3. Comparison of microparameters calculated using invasive and IDIF. a)** Correlation of *k_2_*, **b)** *k_3_* and **c)** *k_4_*, n=15 (6 naive animals and 9 MI animals) with 3 regions per animal (heart, brain and left ventricular anterior wall).


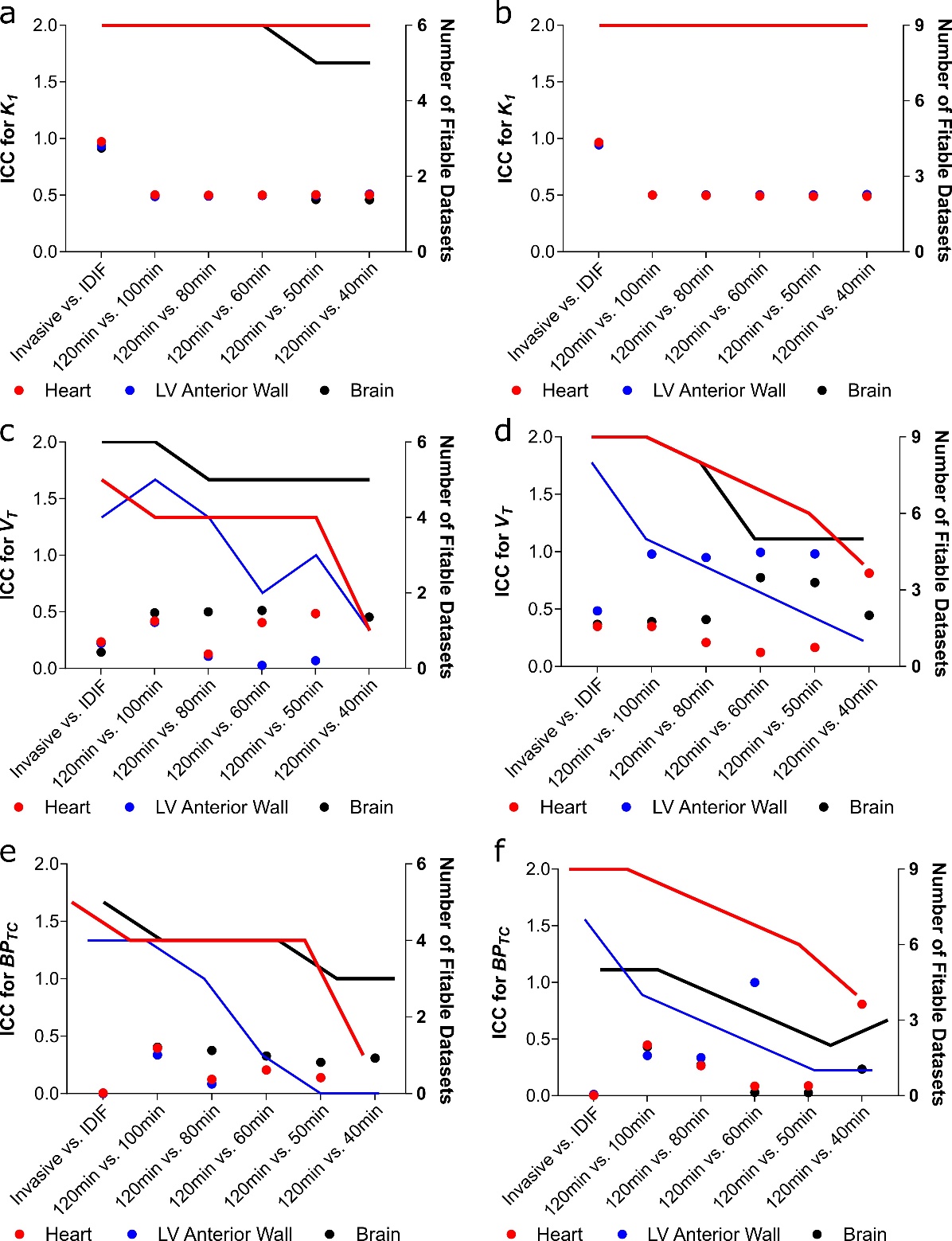


**Supplementary Figure 4. The ICC of 2TCM parameters for invasive input function, IDIF and PET frame truncation in naive and MI cohorts. a)** The ICC (represented as dots) for *K_1_* in naive and **b)** MI cohorts calculated using the different conditions (left Y axis), with the number of subjects (rats) where calculation of *K_1_* was possible shown by the lines (right Y axis). **c)** The same analysis is shown for *V_T_* in the naive and **d)** MI cohorts and **e)** *BP_TC_* in the naive and **f)** MI cohorts. naive n=6, MI n=9.


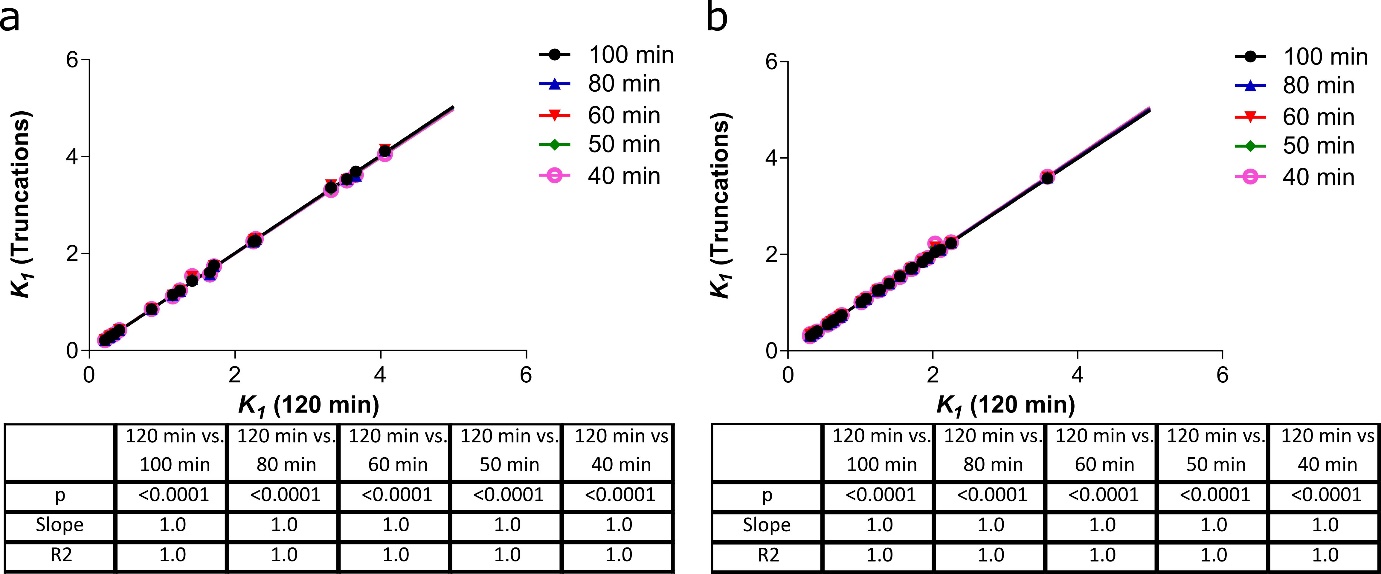


**Supplementary Figure 5. The impact of PET scan duration truncation on the calculation of *K_1_* in both cohorts. a)** Calculation of *K_1_* with different truncations in naive and **b)** MI cohorts, n=6 for naive and n=9 for MI with 3 regions per animal (heart, brain and left ventricular anterior wall).


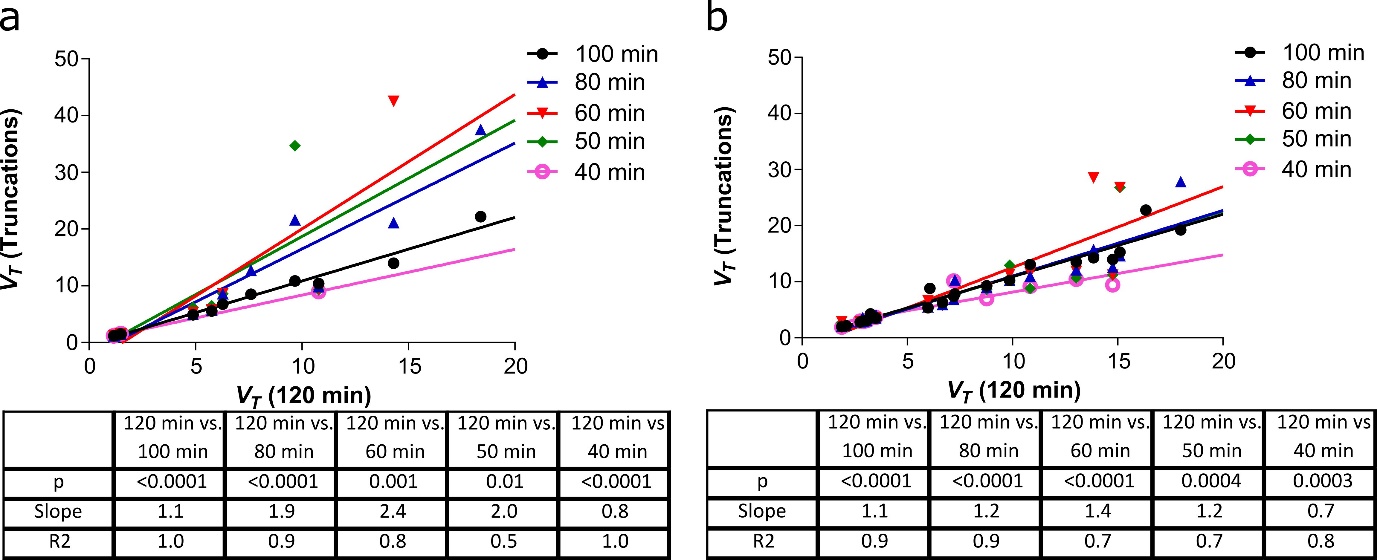


**Supplementary Figure 6. The impact of PET scan duration truncation on the calculation of *V_T_* in both cohorts. a)** Calculation of *V_T_* with different truncations in naive and **b)** MI cohorts, n=6 for naive and n=9 for MI with 3 regions per animal (heart, brain and left ventricular anterior wall).


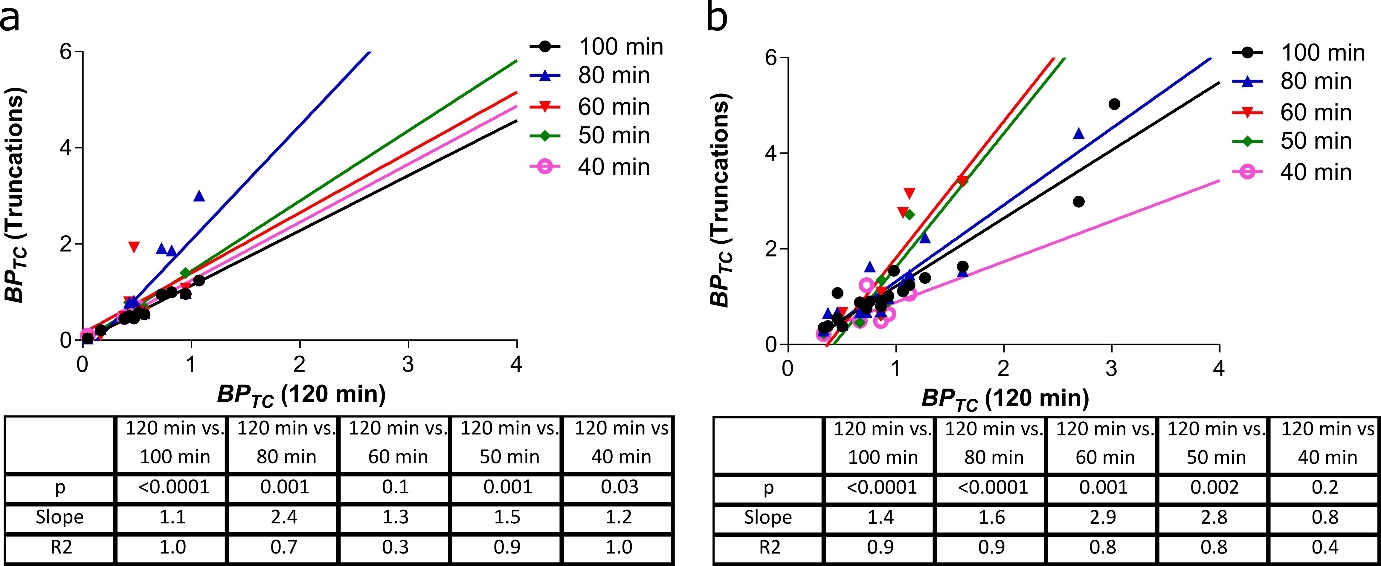


**Supplementary Figure 7. The impact of PET scan duration truncation on the calculation of *BP_TC_* in both cohorts. a)** Calculation of *BP_TC_* with different truncations in naive and **b)** MI cohorts, n=6 for naive and n=9 for MI with 3 regions per animal (heart, brain and left ventricular anterior wall).
